# The impact of transcranial magnetic stimulation on diagnostic confidence in patients with Alzheimer disease

**DOI:** 10.1186/s13195-018-0423-6

**Published:** 2018-09-18

**Authors:** Alberto Benussi, Antonella Alberici, Clarissa Ferrari, Valentina Cantoni, Valentina Dell’Era, Rosanna Turrone, Maria Sofia Cotelli, Giuliano Binetti, Barbara Paghera, Giacomo Koch, Alessandro Padovani, Barbara Borroni

**Affiliations:** 1 Neurology Unit, Department of Clinical and Experimental Sciences, University of Brescia, Brescia, Italy; 2 IRCCS Centro San Giovanni di Dio Fatebenefratelli, Brescia, Italy; 3 Department of Neuroscience, Psychology, Drug Research and Child Health, University of Florence, Florence, Italy; 4 Neurology Unit, Ospedale Vallecamonica, Esine, Brescia, Italy; 5 Nuclear Medicine Unit, Spedali Civili Brescia, Brescia, Italy; 6 Non Invasive Brain Stimulation Unit, IRCCS Santa Lucia Foundation, Rome, Italy; 7 Stroke Unit, Policlinico Tor Vergata, Rome, Italy

**Keywords:** Alzheimer disease, Frontotemporal dementia, Transcranial magnetic stimulation, PET amyloid, Diagnosis, Confidence

## Abstract

**Background:**

Cholinergic dysfunction is a key abnormality in Alzheimer disease (AD) that can be detected in vivo with transcranial magnetic stimulation (TMS) protocols. Although TMS has clearly demonstrated analytical validity, its clinical utility is still debated. In the present study, we evaluated the incremental diagnostic value, expressed in terms of diagnostic confidence of Alzheimer disease (DCAD; range 0–100), of TMS measures in addition to the routine clinical diagnostic assessment in patients evaluated for cognitive impairment as compared with validated biomarkers of amyloidosis.

**Methods:**

One hundred twenty patients with dementia were included and scored in terms of DCAD in a three-step assessment based on (1) demographic, clinical, and neuropsychological evaluations (clinical work-up); (2) clinical work-up plus amyloid markers (cerebrospinal fluid or amyloid positron emission tomographic imaging); and (3) clinical work-up plus TMS intracortical connectivity measures. Two blinded neurologists were asked to review the diagnosis and diagnostic confidence at each step.

**Results:**

TMS measures increased the discrimination of DCAD in two clusters (AD-like vs FTD-like) when added to the clinical and neuropsychological evaluations with levels comparable to established biomarkers of brain amyloidosis (cluster distance of 55.1 for clinical work-up alone, 76.0 for clinical work-up plus amyloid markers, 80.0 for clinical work-up plus TMS). Classification accuracy for the “gold standard” diagnosis (dichotomous - AD vs FTD - variable) evaluated in the three-step assessment, expressed as AUC, increased from 0.82 (clinical work-up alone) to 0.98 (clinical work-up plus TMS) and to 0.99 (clinical work-up plus amyloidosis markers).

**Conclusions:**

TMS in addition to routine assessment in patients with dementia has a significant effect on diagnosis and diagnostic confidence that is comparable to well-established amyloidosis biomarkers.

**Electronic supplementary material:**

The online version of this article (10.1186/s13195-018-0423-6) contains supplementary material, which is available to authorized users.

## Background

The clinical diagnosis of neurodegenerative disorders is based on an extensive evaluation of cognitive and behavioral performance, along with functional status, which provides a variable grade of accuracy, with a definite diagnosis reached only at autopsy [1, 2]. Many technological advancements have been implemented to serve as surrogates for specific neuropathological hallmarks and to improve the diagnostic work-up of dementia [3]. Over the past decade, many steps have been taken to increase the accuracy of Alzheimer disease (AD) diagnosis, and recent criteria state that positivity of one or more biomarkers of brain amyloidosis or neuronal injury is associated with a high likelihood of AD [4, 5]. Furthermore, important innovations in ongoing clinical trials in AD now include the use of preclinical/prodromal biomarkers, considering the increasing evidence that disease-modifying treatments must be administered early in the disease course [6, 7]. As a result, the development of diagnostic tools capable of accurately discriminating AD from frontotemporal dementia (FTD) at early disease stages has become a crucial target [4].

Decreased levels of amyloid-β 1–42 (Aβ_1–42_) in the cerebrospinal fluid (CSF) and/or increased binding of amyloid ligands visualized by positron emission tomography (PET) are the most established and validated amyloid markers [8–11], being helpful in increasing the diagnostic confidence of Alzheimer disease (DCAD) among clinicians [12, 13]. However, despite the usefulness of these markers, a number of drawbacks, such as invasive procedures (i.e., CSF collection), expensiveness (i.e., PET amyloid), or availability restricted to tertiary dementia centers, may limit their use. Concomitantly, there is a growing demand to identify inexpensive, easy-to-perform, noninvasive, and safe biomarkers to be used extensively on clinical grounds [14].

In this context, our group has recently developed an index using transcranial magnetic stimulation (TMS) intracortical connectivity measures, yielding a diagnostic accuracy of 90% in identifying AD with high accuracy even in the early phases of disease [15, 16]. Short-latency afferent inhibition (SAI), assessing the function of cholinergic circuits indirectly, has been found to be impaired in patients with AD [15–17]; conversely, short-interval intracortical inhibition (SICI) and intracortical facilitation (ICF), markers of γ-aminobutyric acid type A (GABA_A_)ergic and glutamatergic neurotransmission, respectively, have been found to be impaired in patients with FTD [17, 18]. These findings stemmed from the evidence that AD is defined by both amyloid deposits and a well-established cholinergic deficit [19–22], whereas in FTD, abnormalities in glutamatergic and GABAergic neurotransmission have been reported [23–26].

The assessment of TMS intracortical connectivity holds promise to be a useful tool in the differential diagnosis of neurodegenerative diseases, being free from strict exclusion criteria, not time-consuming, and inexpensive [27]. However, its clinical value needs to be further demonstrated, also taking into consideration that both conditions may show several overlapping features, such as amyloid positivity in FTD [28], cholinergic deficits in FTD [29], or glutamatergic overexpression in AD [30]. Indeed, several studies now suggest that some cases of AD and FTD are linked in a genetic spectrum of degenerative brain disorders in which tau appears to be the central player [31]. Moreover, this is especially important in patients with late-onset disease, in whom differential diagnosis becomes more challenging owing to overlapping symptoms and mixed neuropathology.

All the above observations defined the objective of this work, aimed at evaluating the clinical utility of TMS compared with amyloid markers in DCAD. To this end, the impact of TMS intracortical connectivity measures was compared with that of amyloid markers when both were added to routine clinical assessment. A validation of DCAD, in terms of prediction performance of a “gold standard” diagnosis, concluded the work.

## Methods

### Approval

Full written informed consent was obtained from all participants according to the Declaration of Helsinki. The study protocol was approved by the local ethics committee (NP1965; approved 19 May 2015).

### Participants and study design

Patients with either probable AD [5] or FTD [32, 33] were consecutively recruited from the Neurology Unit, Department of Clinical and Experimental Sciences, University of Brescia, Italy.

Probable AD was defined as the presence of cognitive or neuropsychiatric symptoms with an insidious onset that interfered with the ability to function at work or at usual activities, involving a minimum of two cognitive domains, with evidence of the AD pathophysiological process [5]. Patients with FTD were diagnosed as behavioral variant FTD (bvFTD) [33] or semantic variant FTD (svPPA) and agrammatic variant of primary progressive aphasia (avPPA), according to current clinical criteria [32]. These criteria have shown good correlations in clinicopathological studies [34, 35].

Demographic characteristics, family history, and clinical features were carefully recorded. During the first visit, dementia experts (MSC, AP, and BB) performed a neurological, cognitive, and behavioral examination and did a preliminary assessment of eligibility. All patients considered in the present study underwent a standardized neuropsychological evaluation, brain magnetic resonance imaging (MRI), at least one biomarker of brain amyloidosis (i.e., CSF Aβ_1–42_ dosage and/or amyloid PET scan), and TMS intracortical connectivity measures, as described below.

Patients with a history of epilepsy or with electronic implants were excluded from the study.

None of the patients were treated with drugs that could have altered the cerebral cortex excitability in the previous 3 months, such as benzodiazepines, acetylcholinesterase inhibitors, neuroleptics, or antidepressants. If patients were already on central nervous system active medications, they were asked to gradually decrease their dosage until suspension, only if this was not accompanied by a relevant deterioration in cognitive, behavioral, or clinical symptoms for which the therapy was restarted, thus excluding the patient from entering the study.

Patients’ data were then anonymized, and the following information was presented to two experienced neurologists (AB and AA) in three separate sections in which they were made aware of (1) demographic characteristics, family history, clinical and neuropsychological assessment, and structural imaging data (henceforth defined as “clinical work-up”); (2) clinical work-up plus amyloid markers; and (3) clinical work-up plus TMS intracortical connectivity measures.

On the basis of the data obtained in section (1), the two experienced neurologists formulated their etiological diagnosis (AD vs FTD) and rated their confidence that cognitive impairment was due to AD on a structured scale ranging from 0% to 100% (DCAD, 0–100%). Thus, DCAD > 50% supported an AD diagnosis, whereas DCAD < 50% supported an FTD diagnosis. In cases in which the same diagnosis was reached by both raters, mean DCAD was considered. In the cases in which there was disagreement (one neurologist suggested AD and the other FTD diagnosis), a second round of joint evaluation was carried out, and a shared diagnosis and DCAD agreement was reached.

The same protocol was adopted for section (2) and section (3), in which the two neurologists were asked to revise patients’ diagnosis and DCAD after disclosure of combined clinical work-up along with either amyloid markers (section 2) or TMS intracortical connectivity measures (section 3). Thus, any change in diagnosis or DCAD in the subsequent sections could only be attributed to the knowledge of such results.

**Fig. 1:**
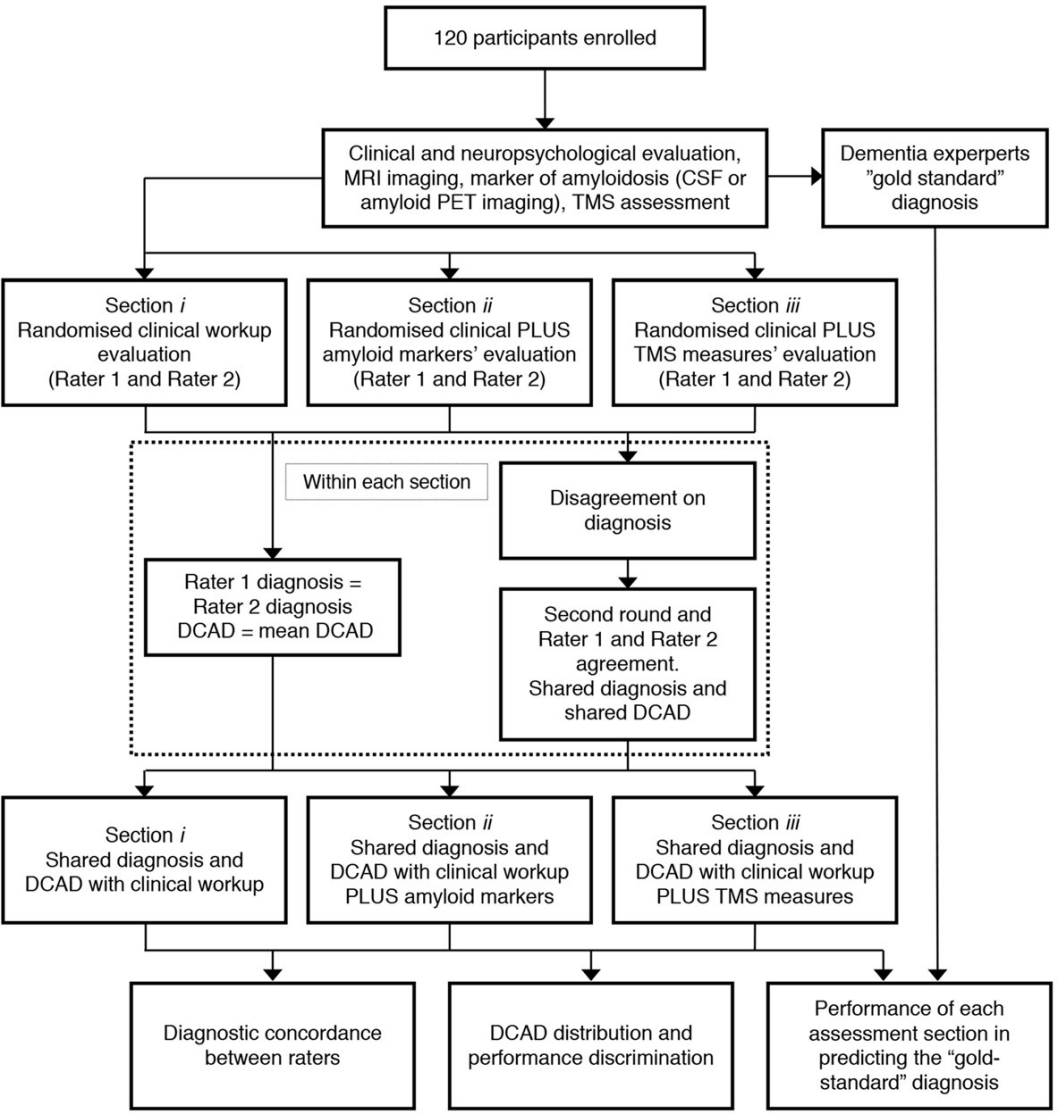
Study design. *CSF* Cerebrospinal fluid, *DCAD* Diagnostic confidence of Alzheimer disease, *MRI* Magnetic resonance imaging, *PET* Positron emission tomography, *TMS* Transcranial magnetic stimulation

In each section, patients were presented in a random order, and for each diagnostic disagreement, a second round of joint evaluation was performed, as for section (1). At the end, a shared diagnosis and mean DCAD between the two raters was provided for each patient regarding clinical work-up, clinical work-up plus amyloid markers, and clinical work-up plus TMS measures (*see* Fig. 1 for study design). Moreover, a “gold standard” diagnosis (i.e., AD or FTD) was provided by the dementia experts (MSC, AP, and BB), who had the patients in charge and who had complete access to all the available information, such as the clinical work-up, amyloid markers, and TMS.

#### Clinical work-up

The set of mandatory information for each recruited subject, which was presented to the two neurologists during the clinical work-up evaluation, included demographic characteristics (age, sex, family history, past medical history, and comorbidities), conventional structural brain imaging findings, and the results of the neuropsychological assessment, including global cognitive function, long-term memory, executive function, and language and visual spatial abilities. In particular, for all patients, at least the following tests were available: Mini Mental State Examination (MMSE) [36], Clinical Dementia Rating [37], short story [38], copy and recall of the Rey-Osterrieth complex figure test [39], Trail Making Test part A and part B [40], semantic and phonemic fluency [41], and clock-drawing test. Moreover, basic [42] and instrumental activities of daily living [43] and the Neuropsychiatric Inventory [44] were reported. All the above data were provided to the two raters in sections (1), (2), and (3).

#### Amyloid markers

In this group, diagnostic markers of amyloidosis, including CSF Aβ_1–42_ analysis or amyloid PET imaging, were assessed. Lumbar puncture was carried out in the outpatient clinic according to standard procedures, and CSF analysis was performed using an enzyme-linked immunosorbent assay (INNOTEST; Innogenetics, Ghent, Belgium) [45]. Accordingly, a CSF AD-like profile was defined as CSF Aβ_1–42_ ≤ 650 ng/L (along with CSF Tau ≥ 400 ng/L). Amyloid PET imaging was acquired using 370 MBq (10 mCi) of ^18^F-florbetapir, and visual readings were performed by a nuclear medicine physician who was blinded to the patients’ diagnoses, following the procedures provided by the ligand manufacturer, as previously reported [12]. CSF Aβ_1–42_ dosage (along with CSF tau) and/or amyloid PET result (amyloid PET “positive” vs “negative”) were provided to the two raters in section (ii).

#### Transcranial magnetic stimulation intracortical connectivity measures

TMS protocols were carried out as previously published [46]. TMS was performed with a figure-of-eight coil (each loop diameter 70 mm) connected to a Magstim Bistim^2^ system (Magstim Company, Oxford, UK). Motor evoked potentials (MEPs) were recorded from the right first dorsal interosseous muscle (FDI) through surface Ag/AgCl electrodes placed in a belly-tendon montage and acquired using a Biopac MP-150 electromyograph (BIOPAC Systems Inc., Santa Barbara, CA, USA). The TMS coil was held tangentially over the scalp region corresponding to the primary hand motor area contralateral to the target muscle, with the coil handle pointed 45 degrees posteriorly and laterally to the sagittal plane. The motor hot spot was defined as the location where TMS consistently produced the largest MEP size at 120% of the resting motor threshold (rMT) in the target muscle.

rMT was defined as the minimal stimulus intensity needed to produce MEPs with an amplitude of at least 50 μV in five of ten consecutive trials during complete muscle relaxation, which was controlled by visually checking the absence of electromyographic (EMG) activity at high-gain amplification [47].

We considered SICI and ICF, which predominantly reflect GABA_A_ergic and glutamatergic neurotransmission, respectively [48, 49], and SAI, which primarily reflects cholinergic transmission, using a previously described technique [50]. SICI and ICF were studied at rest via a paired-pulse paradigm, delivered in a conditioning test design with the conditioning stimulus (CS) set at an intensity of 70% of the rMT, whereas the test stimulus (TS) was adjusted to evoke an MEP of approximately 1 mV peak to peak in the relaxed FDI. Different interstimulus intervals (ISIs) between the CS and TS were employed to investigate preferentially both SICI (1, 2, 3, and 5 ms) and ICF (7, 10, and 15 ms) [48, 49].

SAI, which primarily reflects cholinergic transmission, was studied using a previously described technique [50]. CSs were single pulses (200 μs) of electrical stimulation applied through bipolar electrodes to the right median nerve at the wrist (cathode proximal). The intensity of the CS was set at just over motor threshold for evoking a visible twitch of the thenar muscles, whereas the TS was adjusted to evoke an MEP of approximately 1 mV peak to peak. The CS to the peripheral nerve preceded the TS by different ISIs (− 4, 0, + 4, + 8 ms, determined relative to the latency of the N20 component of the somatosensory evoked potential).

Ten stimuli were delivered for each ISI for all stimulation paradigms, and fourteen control MEPs in response to the TS alone, were recorded for each paradigm in all participants in a pseudorandomized sequence. The amplitude of the conditioning MEPs was expressed as a ratio of the mean unconditioned response. The intertrial interval was set at 5 s (± 10%).

SICI-ICF and SAI protocols were performed in a randomized order. Throughout the experiment, complete muscle relaxation was monitored by audiovisual feedback when appropriate. If the quality of study data was degraded by patient movement, the protocol was recommenced, and the initial data were discarded. Trials were discarded if EMG activity exceeded 100 μV in the 250 ms prior to TMS stimulus delivery. All patients were able to understand instructions and obtain full muscle relaxation.

The SICI-ICF/SAI ratio was defined as average SICI (1, 2, 3 ms)/average ICF (7, 10, 15 ms)/average SAI (0, + 4 ms). The SICI-ICF/SAI ratio was provided to the two raters, and they considered the previous published cutoff value of 0.98 [15] in section (iii). The operators who performed TMS (VC and VD) were blinded to the subjects’ amyloid marker status and clinical/neuropsychological evaluation.

### Statistical analysis

Sociodemographic characteristics of the patients as well as descriptive features of the DCAD were provided through mean, SD, and median values. To assess the correlation and agreement of DCAD between the two raters, and between each rater and the shared DCAD, nonparametric Spearman’s correlation, and intraclass correlation coefficient (ICC; single measures) were used.

A data-driven model-based classification method, namely a Gaussian mixture model [51, 52], was applied to evaluate the performance of the three different assessments (clinical work-up, clinical work-up plus amyloid markers, and clinical work-up plus TMS) in discriminating between AD and FTD. A mixture model procedure allows one to obtain a probabilistic clustering that quantifies the uncertainty of the data belonging to components (clusters) of the mixture. The estimation procedure was carried out using an expectation-maximization algorithm [53], and the number of clusters was estimated on the basis of the Bayesian information criterion (BIC) index for goodness of model fit (the lower the BIC, the better the model fits) [54]. Once clusters for each assessment DCAD distribution were detected through mixture models, two different indices were carried out to evaluate the discriminative performance in each assessment section: the cluster centroid distance (i.e., distance, in terms of DCAD, between the two cluster middles) and the DCAD mean with 95% CI of the mixture clusters. In particular, for the cluster means (i.e., the means of the Gaussian components of each mixture model), statistical difference was evaluated through *t* tests.

Finally, the association of DCAD of each of the three sections (independent variables) with the “gold standard” diagnosis (dichotomous dependent variable) was evaluated through logistic regression models. Performance of each assessment section in predicting the “gold standard” diagnosis was evaluated through ROC curves and the corresponding AUC values applied on predictive probability scores obtained by the logistic models. High values of AUC (> 0.8) indicate good performance of independent variables in predicting the diagnosis and thus in classifying AD vs FTD. Comparison of AUC was performed using the DeLong test.

Statistical significance was assumed at *p* < 0.05. Data analyses were carried out using the ‘mclust’ and ‘InformationValue’ packages of R statistical software (http://www.r-project.org).

## Results

### Participants and TMS measures

Among 120 patients, 57 (47.31%) were female, the mean age was 67.5 ± 8.36 years, the mean age at onset was 64.1 ± 7.90 years, and the mean MMSE score was 23.5 ± 5.93. According to the “gold-standard” diagnosis, 63 patients were classified as AD (mean age, 70.3 ± 7.2; female sex, 52.4%; MMSE score, 22.8 ± 5.6) and 57 as FTD (mean age, 64.4 ± 8.5; female sex, 42.1%; MMSE score, 24.2 ± 6.3). Demographic, clinical, and neurophysiological scores for each group of patients are reported in Table 1. In the FTD group, 42 patients were classified as bvFTD, 8 as avPPA, and 7 as svPPA. No significant differences in average SICI (*p* = 0.161), ICF (*p* = 0.936), or SAI (*p* = 0.678) were observed between FTD subgroups. All procedures, including TMS sessions, were generally well tolerated with no adverse events reported in the whole cohort of patients.

**Table 1:** Demographic, clinical, and neurophysiological characteristics of included patients

	AD	FTD	*p* Value*
Patients (number)	63	57	–
Age, years	70.3 ± 7.2	64.4 ± 8.5	*p* < 0.001
Age at onset (years)	66.9 ± 6.9	60.9 ± 7.8	*p* < 0.001
Sex (% female)	52.4	42.1	n.s.
Education (years)	10.1 ± 4.7	10.2 ± 4.5	n.s.
MMSE	22.8 ± 5.6	24.2 ± 6.3	n.s.
CDR	0.9 ± 0.5	0.9 ± 0.6	n.s.
NPI	10.9 ± 8.4	16.8 ± 10.6	*p* = 0.002
Short story	3.9 ± 3.2	8.3 ± 11.0	*p* = 0.009
Rey figure copy	19.3 ± 11.7	24.8 ± 9.4	*p* = 0.022
Rey figure recall	4.0 ± 5.2	8.2 ± 6.4	*p* = 0.001
TMT-A (s)	179.2 ± 177.5	112.8 ± 136.8	*p* = 0.053
TMT-B (s)	437.8 ± 152.7	345.3 ± 175.4	*p* = 0.008
Phonemic fluency	21.6 ± 9.6	16.6 ± 10.9	*p* = 0.023
Semantic fluency	20.3 ± 12.3	32.5 ± 70.2	n.s.
Clock-drawing test	5.5 ± 2.8	6.7 ± 2.8	*p* = 0.028
BADL lost	0.3 ± 0.9	0.6 ± 1.2	n.s.
IADL lost	1.8 ± 2.2	1.8 ± 2.3	n.s.
Amyloid PET, number positive	38/38	1/7	*p* < 0.001
Cerebrospinal fluid			
t-Tau	788.2 ± 414.5	317.0 ± 177.6	*p* < 0.001
p-Tau_181_	129.4 ± 213.5	44.2 ± 24.1	*p* = 0.027
Aβ_1–42_	520.3 ± 109.6	900.6 ± 291.3	*p* < 0.001
TMS measures			
SICI	0.29 ± 0.11	0.67 ± 0.26	*p* < 0.001
ICF	1.41 ± 0.20	0.87 ± 0.22	*p* < 0.001
SAI	0.84 ± 0.10	0.52 ± 0.12	*p* < 0.001
SICI-ICF/SAI ratio	0.26 ± 0.11	1.67 ± 0.91	*p* < 0.001

*Abbreviations: Aβ*_*1–42*_ Amyloid-β 1–42, *AD* Alzheimer disease, *BADL* Basic activities of daily living, *CDR* Clinical Dementia Rating, *FTD* Frontotemporal dementia, *IADL* Instrumental activities of daily living, *ICF* Average intracortical facilitation (7, 10, 15 ms), *ISI* Interstimulus interval, *MMSE*: Mini Mental State Examination, *n.s.* Not significant, *NPI* Neuropsychiatric Inventory, *PET* Positron emission tomography, *p-Tau*_*181*_ Phosphorylated tau_181_, *SAI* Average short-latency afferent inhibition (0, + 4 ms), *SICI* Average short-interval intracortical inhibition (1, 2, 3 ms), *SICI-ICF/SAI* Average SICI at ISI 1, 2, 3 ms/average ICF at ISI 7, 10, 15 ms/average SAI at ISI 0, + 4 ms, *TMS* Transcranial magnetic stimulation, *TMT-A* Trail Making Test part A, *TMT-B* Trail Making Test part B, *t-tau* Total tau

Demographic and clinical characteristics and neurophysiological parameters are expressed as mean ± SD (unless otherwise specified)

*p Values for independent samples *t* test or Fisher’s exact test, as appropriate

### Diagnostic concordance between raters

A high correlation between the two raters (AA’s diagnosis vs AB’s diagnosis) and between each rater and shared diagnosis (AA’s diagnosis vs shared diagnosis and AB’s diagnosis vs shared diagnosis) was reported in all three sections (Spearman’s correlation coefficient ranges, 0.65 to 0.86 in clinical work-up section; 0.82 to 0.91 in clinical work-up plus amyloid markers; 0.79 to 0.85 in clinical work-up plus TMS). Similarly, high values of ICC (AB’s diagnosis, AA’s diagnosis, and shared diagnosis) were found for clinical work-up (ICC, 0.74), and these increased when clinical work-up plus amyloid markers (ICC, 0.88) or clinical work-up plus TMS (ICC, 0.94) was considered. The high values of correlation and agreement legitimize the use, hereafter, of the shared diagnosis (i.e., the mean DCAD between the two raters) as the main outcome variable for assessing DCAD of each assessment section.

### DCAD distribution and performance discrimination of the three assessment sections

A bimodal distribution was found for DCAD in all three section assessments. The Gaussian mixture model approach identified two clusters (lower BIC indices were obtained by mixture models with two-component clusters) in each of the three DCAD distributions: the FTD-like cluster (characterized by lower DCAD values) and the AD-like cluster (defined by higher DCAD values).

**Fig. 2:**
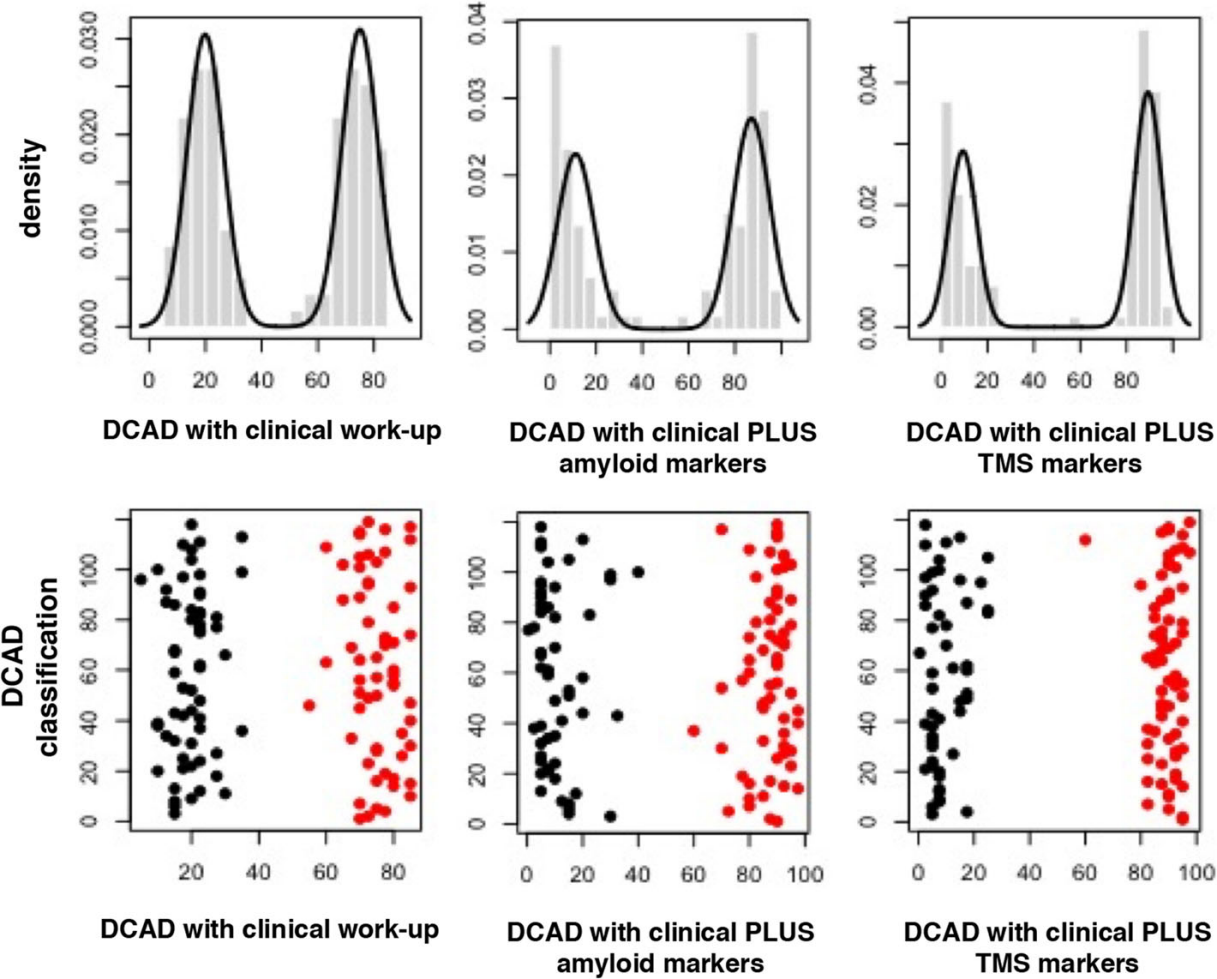
Mixture model estimation (upper panels) and classification (lower panels; *red dots*, Alzheimer disease group; *black dots*, frontotemporal dementia group). *DCAD* Diagnostic confidence of Alzheimer disease, *TMS* Transcranial magnetic stimulation

In Fig. 2, mixture model fitting estimations (Fig. 2a) and corresponding cluster identification (Fig. 2b) are reported. The greater the distance between the two peaks, the higher the discrimination performance between the two diagnoses.

In Table 2, group centroids’ distance of the two DCAD clusters as well as their means and 95% CIs are reported. Although cluster mean differences were statistically different for all the three assessment sections (*p* < 1.0 × 10^− 5^ for all), the lower separation between the two peaks, and thus the lower discrimination performance, was found with clinical work-up (cluster distance equal to 55.1), whereas the separation increased when clinical work-up plus amyloid markers was considered (cluster distance of 76.0) and further increased when clinical work-up plus TMS was analyzed (cluster distance equal to 80.0).

**Table 2:** Performance of the three different assessment sections in discriminating between Alzheimer disease and frontotemporal dementia in terms of diagnostic confidence of Alzheimer disease

Indices of separation	Clinical work-up		Clinical work-up plus amyloid markers		Clinical work-up plus TMS markers	
Cluster mean	Mean (95% CI)	*p* Value	Mean (95% CI)	*p* Value	Mean (95% CI)	*p* Value
AD cluster	75.0 (73.4–76.6)	*p* < 1.0 × 10^− 5^	87.1 (85.2–89.0)	*p* < 1.0 × 10^− 7^	89.3 (87.9–90.7)	*p* < 1.0 × 10^− 10^
FTD cluster	19.9 (18.2–21.6)		11.1 (9.0–13.2)		9.3 (7.6–10.9)	
AD vs FTD cluster centroid distance	55.1		76.0		80.0	

*Abbreviations: AD* Alzheimer disease, *FTD* Frontotemporal dementia, *TMS* Transcranial magnetic stimulation

DCAD mean (and 95% CI) of the two clusters correspond to the means of the two estimated Gaussian components of each mixture model. P-value refers to the difference between cluster means within each assessment

### Performance of each assessment section in predicting the “gold standard” diagnosis

Logistic regression models revealed a high statistically significant association between “gold standard” diagnosis and all the three assessments (*p* < 0.046 for all). In detail, an enhancement of one unit in DCAD value corresponds to an increased probability to reach a diagnosis of AD of 5%, 11%, and 32% (ORs equal to 1.05, 1.11, and 1.32, respectively), for clinical work-up, clinical work-up plus amyloid markers, and clinical work-up plus TMS assessment, respectively.

Considering the performance in predicting the “gold standard” diagnosis, although all three assessments reached high values of specificity and sensitivity in classifying AD vs FTD correctly (AUC > 0.8 for all), the best performance was obtained by clinical work-up plus amyloid markers and by clinical work-up plus TMS measures (*see* Table 3). AUCs of clinical work-up plus amyloid markers and of clinical work-up plus TMS were statistically different from the AUC of clinical work-up (De Long test *p* = 2.6 × 10^− 5^ and *p* = 4.1 × 10^− 5^, respectively), and, interestingly, these were not different from each other (*p* = 0.619) (*see* Additional file 1: Figure S1).

**Table 3:** Association (measured by OR) and performance (measured by AUC) evaluation of diagnostic confidence of Alzheimer disease of each assessment section in predicting the “gold standard” diagnosis

Assessment	AUC (95% CI)	OR (95% CI)	*p* Value
Clinical work-up	0.82 (0.74–0.90)	1.05 (1.03–1.07)	4.9 × 10^−9^
Clinical work-up plus amyloid markers	0.99 (0.98–1)	1.11 (1.08–1.16)	6.1 × 10^−8^
Clinical work-up plus TMS	0.98 (0.96–1)	1.32 (1.11–1.83)	0.046

*TMS* Transcranial magnetic stimulation

*p* Value refers to the statistical significance of ORs

AUC 95% CI: If CI includes 0.5, the AUC is statistically not different from an AUC obtained from a classification by chance

## Discussion

It has been widely demonstrated that the DCAD can be improved by the use of biological markers [12]. A number of biomarkers of functional impairment, neuronal loss, and protein deposition that can be assessed by neuroimaging (i.e., MRI and PET) or CSF analysis have been validated to diagnose AD in research studies and specialist clinical settings so far [3]. Some of these biomarkers have shown very high diagnostic accuracy in discriminating AD from FTD. Indeed, previous studies have shown that fluorodeoxyglucose (FDG)-PET has a diagnostic accuracy of up to 87% in differentiating AD from FTD, amyloid PET imaging has a diagnostic accuracy of 97% [55], and CSF analysis has a diagnostic accuracy of up to 95% [56]. Each biomarker has been proven reliable and accurate, even though a number of drawbacks might limit its use in clinical settings. FDG-PET, which provides important information on the topographical distribution of the ongoing neurodegenerative process and thus limited in the differential diagnosis of focal variants of AD, is still an expensive procedure. Amyloid PET imaging, which provides pathophysiological information such as the accumulation of Aβ plaques, besides being expensive, is still not available in all dementia centers. CSF analysis, which provides pathophysiological information on both tau and Aβ accumulation, is an invasive procedure with an albeit low but possible risk of complications and cannot be performed in patients on anticoagulation therapy. In this study, we have shown that TMS assessment added to the clinical and neuropsychological evaluation may increase diagnostic accuracy up to 98%, comparable to that obtained from the addition of amyloidosis biomarkers, which reached 99% accuracy.

Concurrently, in the aging population, the prevalence of AD and other neurodegenerative dementias is steadily increasing worldwide [1], thus making it crucial to find accurate but inexpensive markers that can be used to screen at-risk populations. Furthermore, ideal markers should be easy to measure and noninvasive to be implemented in secondary referral centers.

In this work, we confirmed previous literature data suggesting that the use of biomarkers increased DCAD in clinical settings as compared with clinical and neuropsychological evaluation alone [12], and we proposed TMS intracortical connectivity measures as a noninvasive diagnostic tool to be added to the clinical work-up.

TMS intracortical connectivity measures may evaluate the other side of the coin of AD pathophysiology: Instead of targeting amyloid deposition by measuring CSF Aβ_1–42_ or binding of amyloid brain imaging ligands on PET, TMS measures assess neurotransmitter deficits, namely the well-established impairment in cholinergic transmission observed in AD [27, 57].

Another well-established hallmark of AD is the impairment in synaptic plasticity observed in animal models of disease [58]. Concurrently, in vivo correlates of long-term potentiation (LTP)-like plasticity, evaluated with paired associative stimulation (PAS) protocols [59] or repetitive TMS [57, 60], have shown a significant impairment of LTP-like cortical plasticity in patients with AD. These studies have been limited to the evaluation of the motor cortex, however, which is probably not the most affected brain region in AD. This restriction has been exceeded by using TMS-electroencephalogram coregistration techniques in brain regions other than the motor cortex, such as the dorsolateral prefrontal cortex [61] or precuneus [62], confirming previous findings. However, in FTD, few studies have also shown a significant impairment in LTP-like plasticity evaluated with PAS in both genetic and sporadic FTD [18, 63]. It is still debated if plasticity protocols might aid in the discrimination between FTD and AD, and specific studies are currently lacking.

Some limitations of the present study need to be acknowledged. First, this is a monocentric study conducted in a tertiary referral center, and we evaluated the differential diagnosis of only two neurodegenerative disorders. Multicenter studies, including secondary referral centers and considering a broader spectrum of neurodegenerative dementias, are needed. Moreover, we conducted a retrospective study using medical records; thus, the evaluation of the add-on TMS parameters’ value in DCAD should be addressed further in real-world situations.

## Conclusions

Our data suggest that TMS measures increase discrimination performance when added to the clinical and neuropsychological evaluation with levels comparable to those of established biomarkers of brain amyloidosis. If these results were corroborated in larger samples, including subjects with mild cognitive impairment, TMS intracortical connectivity measures hold promise to be considered a helpful screening marker to be added to currently available diagnostic tools. Further studies considering both accuracy and economic burden of potential biomarkers are warranted.

## Additional file

Additional file 1: **Figure S1.** ROC curves for clinical work-up, clinical work-up plus amyloid markers, and clinical work-up plus TMS. *TMS* Transcranial magnetic stimulation. (TIF 428 kb)
