# Supplementary figures and images for: The impact of transcranial magnetic stimulation on diagnostic confidence in patients with Alzheimer disease
Source: Alzheimers Res Ther. 2018 Sep 18;10:94. doi: 10.1186/s13195-018-0423-6 (PMC6145195; doi:10.1186/s13195-018-0423-6)

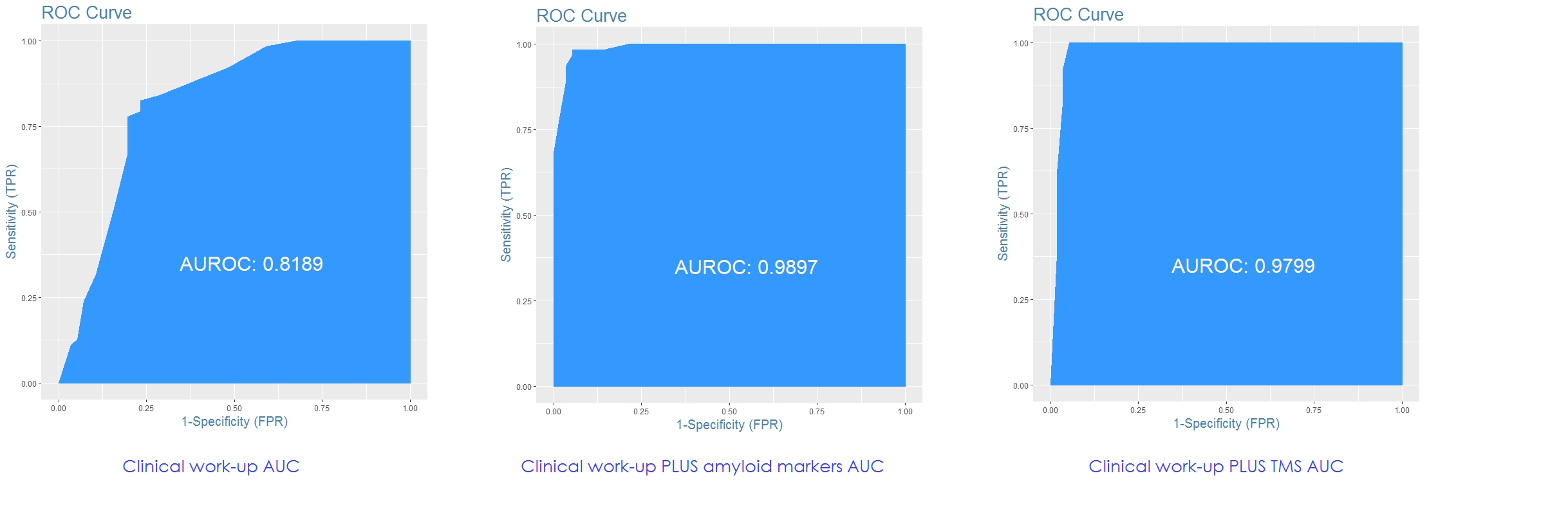

Supplement: Supplementary file 1 — Figure S1. ROC curves for clinical work-up, clinical work-up plus amyloid markers, and clinical work-up plus TMS. TMS Transcranial magnetic stimulation. (TIF 428 kb) [file 13195_2018_423_MOESM1_ESM.tif]
